# Yeast as a Model for Human Disease

**DOI:** 10.3390/ijms27041632

**Published:** 2026-02-07

**Authors:** Bartłomiej Zieniuk, Katarzyna Wierzchowska, Karina Jasińska, Joanna Kobus, Aleksandra Piotrowicz, Şuheda Uğur, Agata Fabiszewska

**Affiliations:** Department of Chemistry, Institute of Food Sciences, Warsaw University of Life Sciences-SGGW, 159C Nowoursynowska Str., 02-776 Warsaw, Poland; bartlomiej_zieniuk@sggw.edu.pl (B.Z.); suheda_ugur@sggw.edu.pl (Ş.U.)

**Keywords:** humanization, neurodegeneration, mitochondria, oncology, pharmacology, genomics, SARS-CoV-2

## Abstract

Yeasts, especially the conventional species *Saccharomyces cerevisiae* and *Schizosaccharomyces pombe*, as well as some unconventional species such as *Pichia pastoris*, *Kluyveromyces marxianus* and *Yarrowia lipolytica*, have become fundamental model organisms for understanding the molecular mechanisms underlying human diseases. Their eukaryotic cell organization, genetic simplicity, and strong conservation of essential biological pathways make them indispensable in biomedical research. This review provides a comprehensive overview of the role of different yeast species in modeling human disorders, highlighting historical milestones and groundbreaking discoveries that have shaped current knowledge. The article discusses the applications of yeast models in studying neurodegenerative diseases such as Alzheimer’s and Huntington’s, as well as metabolic diseases, infectious diseases and mitochondrial disorders, and their growing importance in cancer research and drug discovery. Special attention is given to humanized yeast models, which enable the expression and functional analysis of human genes and the heterologous synthesis of human proteins within yeast cells. Finally, the paper addresses the limitations and challenges of yeast as a model system while outlining future directions and emphasizing the organism’s continued relevance in personalized medicine and functional genomics.

## 1. Introduction

Model organisms are indispensable tools in biological research, enabling the discovery of fundamental principles of genetics and conserved cellular and molecular processes [1]. The concept dates back to Gregor Mendel, who chose *Pisum sativum* (garden pea) for his classic experiments on heredity [2]. Since then, a small number of organisms have become central reference systems: *Drosophila melanogaster* for genetics [3], *Caenorhabditis elegans* for developmental biology [4], *Arabidopsis thaliana* for plant research [5], and *Mus musculus* for mammalian genetics and disease modeling [1]. These species provide experimentally tractable and broadly applicable frameworks for studying life processes.

Among eukaryotic models, yeast occupies a unique position. *Saccharomyces cerevisiae*, a single-celled fungus long used by humans to produce bread, beer, and wine, became a scientific model only after the pioneering work of Louis Pasteur, who demonstrated that fermentation is mediated by living microorganisms [6], and Emil Christian Hansen, who isolated pure yeast cultures at the Carlsberg Laboratory. The foundations of yeast genetics were later established by Øjvind Winge in the 1930s, who introduced Mendelian analysis and demonstrated controlled mating and sporulation. This groundwork allowed subsequent breakthroughs in chromosome mapping, recombination, and transformation [1].

The power of yeast as a model organism lies in its combination of simplicity and deep evolutionary conservation. *S. cerevisiae* can be maintained as stable haploid or diploid strains and is highly amenable to genetic manipulation, including homologous recombination and targeted gene integration [6]. At the same time, many core cellular processes are conserved between yeast and humans. Systematic “humanization” experiments revealed that nearly half of the essential yeast genes with identifiable orthologs can be functionally replaced by their human counterparts despite a billion years of divergence [7]. Importantly, this functional compatibility extends beyond individual genes to higher-order regulatory networks, as key pathways such as AMPK/Snf1 and the Tor signaling network are also strongly conserved [8].

These properties have enabled discoveries that have been recognized with multiple Nobel Prizes. Leland Hartwell and Paul Nurse elucidated the genetic control of the eukaryotic cell cycle using *S. cerevisiae* and *Schizosaccharomyces pombe*, respectively [9,10,11,12]. Randy Schekman uncovered the molecular machinery of vesicle trafficking [13], and Yoshinori Ohsumi defined the mechanisms of autophagy [14]. Other yeast-based studies have revealed the function of telomeres [15], the molecular basis of eukaryotic transcription [16], and ubiquitin-mediated protein degradation [17]. These landmark contributions underscore the central role of yeast in modern cell biology and cancer research [9,18].

The complete sequencing of the *S. cerevisiae* genome in 1996 marked a turning point, propelling yeast to the forefront of functional genomics and systems biology [7,18]. Yeast has become a proving ground for some genetic tools, such as genome-wide deletion libraries [19], high-throughput methods such as synthetic genetic array (SGA) analysis [20], and global transcriptional profiling [21]. More recently, CRISPR/Cas9 editing, single-cell transcriptomics, and proteome-wide interaction mapping have further expanded its utility [8,22]. When the disease-associated genes in humans lack close orthologs in yeast, expressing the human genes associated with diseases in yeast is known as “humanized yeast.” Humanized yeast platforms now enable the systematic testing of disease-associated variants, providing functional insights into rare human genetic disorders [23].

Recent advances continue to extend the relevance of yeast in both basic and translational research. Modern humanized yeast platforms enable the large-scale replacement of yeast genes with human orthologs to assess the function of patient-derived variants and to study protein–protein interactions within conserved pathways [8,23]. Synthetic biology approaches have produced strains optimized for therapeutic applications, such as vaccine antigen production, biosensor development, and metabolic engineering for complex drug precursors [24,25]. Studies on thermotolerance, adaptive evolution for alternative carbon sources, and machine-learning-guided strain optimization illustrate the versatility of yeast as both a discovery platform and an industrial cell factory [26,27].

Despite its simplicity and lack of multicellular structures, the evolutionary conservation of key pathways makes *S. cerevisiae* an essential bridge between unicellular models and complex metazoans [28]. Ongoing innovations—from genome synthesis projects such as Sc2.0 [29] to the integration of high-throughput omics and computational modeling [22]—ensure that yeast remains a premier eukaryotic model for both fundamental research and translational biotechnology.

In this article, we provided a comprehensive overview of yeast as a model organism for studying human diseases. We compared the genetic and experimental features of, among others, *S. cerevisiae*, *S. pombe*, and emerging systems such as *Yarrowia lipolytica*. We examined how these models were applied to investigate neurodegenerative disorders, infectious diseases, metabolic and mitochondrial diseases, cancer biology, and drug discovery, with special emphasis on the rapidly growing field of humanized yeast models. By integrating recent technological advances with a critical assessment of current limitations and future opportunities, this review aims to guide researchers seeking to exploit yeast as a versatile and cost-effective platform for elucidating the molecular basis of human pathologies and for accelerating the development of therapeutic strategies.

## 2. Humanized Yeast Models

Despite over a billion years of separate evolution and profound differences in complexity, yeast and humans share thousands of genes that encode proteins performing essential cellular functions [8]. The human genome contains about 20,000 protein-coding genes, while the yeast genome (*S. cerevisiae*) has around 6000. Interestingly, a comparison of the two species revealed that approximately 2100 ortholog groups were identified, including roughly 2300 genes from yeast and 3900 genes from humans [30]. Moreover, yeasts and humans share many functional pathways essential for eukaryotic cell biology, including the cell cycle and programmed cell death, metabolism, protein folding and degradation, as well as other key signaling pathways [31]. Yeast “humanization” is defined as the process of introducing changes or modifications in yeast to make it more comparable to human cells [32]. In detail, humanized yeast refers to strains of yeast that have been genetically engineered to express human genes, synthesize heterologous human proteins, or reflect the course of human metabolic pathways. These modified yeast strains are used as model systems to study human biology, disease mechanisms, and evolutionary processes.

The concept of humanizing yeast originated in 1985 [33]. When the human *RAS* gene was expressed in yeast mutants lacking the *RAS* gene (rasΔ), the human Ras protein was shown to function effectively in yeast. This successful demonstration paved the way for decades of research into humanized yeast models. Laurent et al. [30] outlined five degrees of humanization, ranging from studying non-humanized yeast to expressing human proteins in yeast, humanizing specific amino acids, replacing entire yeast genes with their human counterparts, and even introducing entire human pathways or protein complexes.

Yeast has served as a model for various neurological disorders. Nonetheless, Huntington’s (HD), Parkinson’s (PD), and Alzheimer’s (AD) diseases have been investigated using humanized yeast platforms, which involve expressing human protein genes that lack yeast orthologs. A detailed discussion of these diseases will follow in subsequent sections of this review. Moreover, other conditions, such as Amyotrophic Lateral Sclerosis, Friedreich’s Ataxia, Batten disease, Niemann–Pick disease, and Hereditary Spastic Paraplegia, have been modeled in yeast [34]. Many studies have revealed that humanized yeast models are valuable for studying the functions of proteins and cellular pathways involved in the misfolding, aggregation, and toxicity of proteins linked to neurodegenerative disorders. These models have reinforced connections between oxidative stress, mitochondrial dysfunction, and apoptosis, highlighting conserved mechanisms of protein-misfolding-related cell death across eukaryotes [34].

Numerous authors have focused on producing therapeutic glycoproteins in yeast. However, their use is limited because yeast’s native high-mannose glycosylation is incompatible with human applications. This leads to a shorter in vivo half-life for the proteins, potentially reducing their efficacy and increasing their immunogenicity [35,36]. Over the last two decades, various approaches have been explored to humanize yeast-derived glycoproteins, including enzymatic modifications in vitro and the modulation of host glycosylation pathways in vivo [35,36]. However, notable advances in the glycoengineering of the yeast *Pichia pastoris* have led to the successful production of fully humanized sialylated glycoproteins [37,38]. These developments challenge the traditional dominance of therapeutic protein production based on mammalian cell culture and offer promising new avenues for the effective use of yeast in producing therapeutic glycoproteins for human use. Moreover, the CRISPR-Cas9 system has been successfully utilized to humanize the glycosylation pathways in *Kluyveromyces marxianus* by disrupting the *och1* and *alg3* (mannosyltransferase) genes [39]. Strategies to produce humanized glycosylated end-products were also explored using these yeast species: *Hansenula polymorpha*, *Yarrowia lipolytica*, *S. pombe*, *K. lactis*, *C. albicans*, and *Zygosaccharomyces cidri* [40].

Other remarkable discoveries using humanized yeast include the first successful humanization of skeletal muscle glycolysis in yeast, paving the way to investigate human glycolysis within yeast [41]; the replacement of 414 essential yeast genes with their human orthologs, of which 47% were successfully humanized, a breakthrough that simplifies drug discovery and accelerates studies on entire human processes and pathways [8]; humanization of nucleosomes in *S. cerevisiae* [42]; and elaboration of a yeast platform for testing inhibitors of human proteins useful in cancer research [43].

The future of humanizing yeast looks promising. One of the most exciting prospects is the development of personalized strains [44], in which yeast is engineered to express patient-specific human genes or disease-associated variants, enabling functional assessment of individual mutations. Humanizing yeast also facilitates the study of human diseases in a more ethical and accessible manner. These yeast-based models could speed up discoveries and offer insights that traditional methods might find challenging or impossible to achieve. Utilizing CRISPR and other gene-editing technologies, researchers can accurately alter yeast genomes, crafting strains that not only replicate aspects of human biology but also optimize certain cellular functions for experimental study, thereby enabling more efficient investigation of disease mechanisms [45,46]. The potential uses are extensive, ranging from tackling global health issues to addressing environmental challenges, all while redefining how mankind leverages the power of life itself.

## 3. Yeast in Infectious Disease Research

Yeast, particularly *S. cerevisiae*, serves as a powerful eukaryotic model organism for dissecting mechanisms of infectious diseases due to its genetic tractability and conserved cellular pathways (Table 1). This chapter explores its applications in viral protein function studies and immune modulation, excluding receptor-mediated signaling in inflammation and SARS-CoV-2 drug screens as specified [47,48].

### 3.1. Viral Protein–Host Interactions

Yeast models facilitate the study of viral proteins from pathogens such as SARS-CoV-2 by enabling their expression in a simplified cellular environment. In this context, protein interaction mapping can be performed using yeast two-hybrid systems and proteome-wide screens to reveal how SARS-CoV-2 proteins associate with host factors and disrupt key cellular processes, such as vesicle trafficking, without dependence on mammalian inflammatory receptors [49].

At the same time, yeast display techniques support variant analysis by allowing researchers to evaluate receptor-binding domain (RBD) variants of the SARS-CoV-2 spike protein, identify adaptive mutations, and determine their functional consequences [47,50]. Importantly, the usefulness of yeast extends beyond SARS-CoV-2, as these platforms also contribute to broader virus research by modeling conserved virus–host dynamics that may apply to multiple infectious agents [48]. Overall, these approaches take advantage of yeast’s rapid growth and amenability to mutagenesis to efficiently generate hypotheses that can later be tested in more complex pathogen and host systems.

### 3.2. Immune Modulation by Yeast Components

Yeast-derived components can modulate immune responses in ways that are highly relevant to infectious disease settings, and these effects can occur independently of specific mammalian inflammatory signaling receptors [51,52].

For example, yeast vacuoles have been shown to reduce LPS-induced inflammation in macrophages by influencing the NF-κB pathway, which points to potential therapeutic applications in infection-associated inflammatory responses [51]. In addition to this systemic immunomodulation, yeast-based materials are also useful in intestinal inflammation models, because cell wall extracts can shift cytokine profiles and affect gut barrier function in a manner that mimics how microbes shape mucosal immunity during infection [52]. Moreover, research linked to inflammatory bowel disease (IBD) indicates that certain yeast species can provoke altered immune reactions in the gastrointestinal tract, emphasizing that yeasts may act as environmental contributors to chronic, infection-like inflammatory conditions [53]. Taken together, these findings highlight a dual role for yeast: it serves not only as a convenient experimental model but also as a biologically active modulator capable of shaping immune outcomes.

By combining powerful genetic tools, conserved eukaryotic pathways, and scalable assays in a non-mammalian system, yeast provides an efficient bridge from mechanistic discovery to translational applications in infectious disease.

## 4. Application of Yeasts in Neurodegenerative Diseases

Neurodegenerative diseases, including AD, PD, HD, and amyotrophic lateral sclerosis, form a complex group of debilitating conditions that impact the nervous system. These diseases lead to a progressive decline in neuronal function, resulting in cognitive, motor, and behavioral impairments [54,55,56]. Several underlying pathological mechanisms, such as protein misfolding, mitochondrial dysfunction, and oxidative stress, mark these disorders. These mechanisms can lead to neuronal cell death and the development of symptoms [54]. While yeast cells are notably less complex than human neurons, the fundamental metabolic pathways involved in neurodegeneration are highly conserved in *S. cerevisiae*. They can be extensively utilized to study the pathogenic effects of proteins linked to HD, PD, and AD [57,58].

### 4.1. Alzheimer’s Disease

The most prevalent neurodegenerative illness in the world, AD is responsible for between 60 and 70% of dementia cases. According to estimates, 50 million individuals worldwide currently suffer from this crippling chronic illness, and by 2050, that figure may increase to over 106 million, primarily as a result of an aging population. The irreversible and progressive deterioration of neuronal structures and functioning in key brain regions, such as the hippocampus and neocortex, is a cellular characteristic of AD. Cognitive decline and dementia are the outcomes of this deterioration. Strong experimental evidence suggests that synaptic disruption occurs early in the course of the disease, altering connectivity within brain circuits essential for memory formation and other cognitive activities, such as thinking and comprehension. Consequently, damage to these brain areas leads to memory impairment, challenges with language, and difficulties in learning, which are typically observed during the early clinical stages of AD. The buildup of misfolded proteins into intracellular and extracellular aggregates, made up of protein Tau or Aβ peptides, respectively, is linked to neuronal injury. It is currently unclear if the existence of these two characteristics causes AD or if a series of biological processes, including oxidative stress, mitochondrial malfunction, and apoptosis, is primarily to blame. In any case, it is still unclear how precisely these proteins harm neurons [59,60,61].

The most prominent microorganism used in studies is *S. cerevisiae*, which has been extensively analyzed, and a wealth of genomic data is available [62,63,64]. Additionally, unconventional yeasts such as *S. pombe*, *C. glabrata*, *K. lactis*, and *Y. lipolytica* [65,66,67] have garnered interest as models for AD. Recent research has focused on understanding the mechanisms associated with autosomal dominant mutations in the amyloid precursor protein (*APP*) gene and in the presenilin-1 and presenilin-2 genes (*PSEN1* and *PSEN2*) linked to a small percentage of AD cases. Therapeutic strategies aim to inhibit the production and aggregation of neurotoxic amyloid-beta (Aβ) peptides, as it is believed that mature amyloid fibrils are the toxic form of the protein responsible for the disease. However, growing evidence suggests that intermediate oligomers of Aβ may be the cytotoxic species implicated in neurodegeneration [68,69].

Yeast can heterologously produce Aβ peptides or APP. Both α- and β-secretase activities have been observed in yeast despite the absence of orthologs for human secretases. It has been suggested that the yeast proteases Yap3p and Mkc7p exhibit α-secretase activity. When APP-based substrates and human γ-secretase were co-expressed in *S. cerevisiae*, γ-secretase activity was successfully restored, leading to the generation of Aβ40, Aβ42, and Aβ43 peptides [70,71,72,73]. Miller-Fleming et al. [73] demonstrated that although yeast does not naturally contain β- and γ-secretase, it can produce these enzymes by expressing *PSEN1* and *PSEN2* (encoding presenilin-1 and presenilin-2), *NCSTN* (encoding nicastrin), *APH1A* (encoding secretase subunit) and *PSENEN* (gene encoding presenilin enhancer). This capability allows further research on the enzymes involved and the in vivo production of Aβ42 aggregates. In 2007, von der Haar et al. [68] developed a new practical instrument for studying the in vivo aggregation of Aβ peptides within an organism, using the yeast *S. cerevisiae* as a model system. They found that when the prion-forming domain of the protein Sup35, associated with the endogenous prion [PSI+], is removed, it cannot aggregate. However, when Aβ peptide sequences are substituted for the original prion domain, the protein’s aggregation capability is restored. Importantly, the resulting aggregates have distinct properties, such as susceptibility to detergents and reliance on transacting factors that are typically required to propagate [PSI+] prions [68].

One alternative approach is to explore the modifiers of Aβ toxicity through a yeast-based screening method. An example of such a modifier is latrepirdine (Dimebon™), an antihistamine that has demonstrated potential benefits in clinical trials for neurodegenerative diseases marked by the accumulation of misfolded proteins. Latrepirdine has been shown to promote the removal of α-synuclein protein aggregates in vivo. A key pathway for eliminating aggregated or misfolded proteins is the autophagy–lysosomal pathway, which has been implicated in the pathogenesis of AD. Enhancing this pathway has shown therapeutic potential in AD and other proteinopathies. Researchers utilized the yeast model *S. cerevisiae* and discovered that latrepirdine boosts autophagy while reducing Aβ42 aggregates. The study found that latrepirdine significantly decreased GFP-Aβ42 levels in wild-type strains compared to atg8Δ mutants, attenuating Aβ42-induced toxicity in wild-type cells. These findings suggest a novel mechanism through which latrepirdine induces autophagy and lowers intracellular levels of GFP-Aβ42 [74].

Research has primarily focused on the role of Aβ in the pathogenesis of AD. However, significant attention has recently been given to MAPT (encoding Tau) research, particularly after the discovery of several MAPT mutations in frontotemporal dementia with Parkinsonism linked to chromosome 17 (FTDP-17). Evidence suggests that Tau is essential for Aβ-mediated neurotoxicity, as demonstrated in both in vitro and in vivo studies. Consequently, there are currently very few published research articles that explore the pathogenic characteristics of protein Tau using the yeast *S. cerevisiae* as a model organism [75,76,77].

Neurodegenerative tauopathies, such as AD, are characterized by the hyperphosphorylation and aggregation of tau proteins. In a study by Vandebroek et al. [76], it was shown that human tau produced in yeast acquired pathogenic phosphoepitopes, leading to aggregate formation and a pathological conformation. The yeast kinases Mds1p and Pho85p—orthologs of GSK-3β and Cdk5, respectively—were found to play a role in these processes. Specifically, the inactivation of Pho85 enhanced phosphorylation, conformational changes, and aggregation of tau-4R, the isoform of human tau containing four microtubule-binding repeats. The yeast model hP-tau/MC1, expressing pathological human tau recognized by the MC1 antibody, was also found to accelerate tau aggregation. This model helps isolate altered tau proteins and provides insights into the molecular changes involved in tauopathies. It could also be valuable in identifying substances and genes that affect these mechanisms.

### 4.2. Parkinson’s Disease

PD is a debilitating neurodegenerative disorder characterized by the progressive loss of dopaminergic neurons in the substantia nigra region of the brain, leading to motor and non-motor symptoms such as muscle stiffness, bradykinesia (delayed movements), resting tremors, and postural instability [78,79]. A hallmark of PD pathology is the presence of Lewy bodies (LBs), which contain molecular chaperones, components of the proteasomal and lysosomal degradation systems, and aggregated α-synuclein [80,81]. LBs are thought to act as a cellular defense mechanism, sequestering toxic α-synuclein oligomers that could otherwise contribute to neuronal dysfunction and death [82]. While approximately 10% of PD cases are linked to familial genetic mutations, most occur sporadically with no known hereditary basis [73,83].

Yeast has emerged as a powerful model for studying the role of α-synuclein aggregation and toxicity. The budding yeast *S. cerevisiae* and the fission yeast *S. pombe* have been extensively utilized to investigate how α-synuclein disrupts essential cellular pathways, including vesicular trafficking, protein degradation and mitochondrial function [84,85]. Yeast cells producing human α-synuclein exhibit key pathological features observed in PD, such as the formation of cytoplasmic inclusions and the induction of conserved cellular stress-response pathways [86]. These models have provided crucial insights into how α-synuclein impairs endoplasmic reticulum (ER)-to-Golgi trafficking, leading to cellular dysfunction and toxicity [87].

Moreover, yeast-based studies have been instrumental in identifying genetic modifiers and molecular chaperones that influence α-synuclein aggregation and toxicity [88]. For example, screens in *S. cerevisiae* have revealed genes involved in lipid metabolism, vesicle transport, and protein quality control that either exacerbate or alleviate α-synuclein-induced stress [89]. Additionally, yeast models have facilitated the characterization of PD-associated proteins, such as ATP13A2 (also known as PARK9), which has been shown to modulate α-synuclein toxicity by regulating metal ion homeostasis [87].

Beyond genetic studies, yeast has also been used for high-throughput screening of potential therapeutic compounds that could mitigate α-synuclein toxicity [88]. These screens have identified small molecules that enhance proteostasis, stabilize cellular homeostasis, and reduce oxidative stress, offering promising avenues for drug development [89].

### 4.3. Huntington’s Disease

HD is a devastating neurodegenerative disorder caused by an expansion of CAG repeats in the huntingtin (*HTT*) gene, leading to the production of mutant huntingtin protein with elongated polyglutamine (polyQ) tracts [90]. The accumulation of these mutant proteins results in cellular toxicity, aggregation, and widespread neuronal dysfunction [91]. Despite extensive research, effective therapeutic interventions remain elusive [92,93].

Researchers have successfully engineered yeast strains to express human huntingtin protein fragments with expanded polyQ repeats, which mimic the aggregation and toxicity observed in human HD pathology [94]. These models have enabled the study of protein misfolding, intracellular trafficking, and the cellular stress responses associated with mutant huntingtin expression [91]. Furthermore, *S. cerevisiae* has been instrumental in large-scale genetic screens to identify modifiers of huntingtin toxicity, revealing critical pathways such as autophagy, chaperone-mediated protein folding, and proteasomal degradation [95,96]. Using yeast models, researchers have also identified chemical compounds capable of reducing huntingtin aggregation, paving the way for potential therapeutic approaches. Additionally, mitochondrial dysfunction and oxidative stress, two hallmarks of HD pathology, have been extensively investigated in yeast, providing insight into how mutant huntingtin disrupts cellular respiration and energy metabolism [91].

Although *S. cerevisiae* is the primary yeast model for HD research, other yeast species have also been explored to provide complementary insights into different aspects of the disease. *S. pombe*, a fission yeast, has been employed to study how huntingtin aggregation affects nuclear-cytoplasmic transport and RNA metabolism, both of which are significantly disrupted in neurodegenerative diseases [92]. Unlike *S. cerevisiae*, *S. pombe* has a different mode of cell division and stress response pathways, making it a valuable alternative for investigating cellular responses to protein misfolding [93]. Studies in *S. pombe* have also helped to validate genetic modifiers identified in budding yeast, confirming their relevance in HD pathology [97].

Another yeast species, *P. pastoris*, has been utilized primarily for recombinant protein expression, allowing for large-scale production of mutant huntingtin fragments [93]. This methylotrophic yeast has proven useful for structural studies and biochemical analyses of huntingtin aggregates [94]. Unlike *S. cerevisiae*, *P. pastoris* can perform more complex post-translational modifications, making it valuable for studying the biochemical properties of mutant huntingtin in a eukaryotic system [90]. By producing polyQ-expanded huntingtin fragments, researchers have been able to examine the structural conformation of aggregates, which aids in the development of potential aggregation inhibitors [96].

Emerging research has also explored *K. lactis* as a model for HD. This yeast species has unique metabolic properties and stress response mechanisms that differ from *S. cerevisiae*, making it a useful system for studying mitochondrial dysfunction in HD [91]. As mitochondrial impairment is a critical aspect of neurodegenerative disorders, *K. lactis* provides a distinct platform for examining how mutant huntingtin affects energy production and cellular respiration [93]. Furthermore, studies in *K. lactis* have contributed to our understanding of how huntingtin aggregation influences chromatin organization and cell cycle regulation, two processes implicated in HD progression [95].

Yeast models have significantly advanced our understanding of neurodegenerative diseases, providing a powerful and versatile platform for investigating disease mechanisms and identifying potential therapeutic targets. *S. cerevisiae* and other yeast species have been instrumental in elucidating the cellular processes underlying AD, PD and HD, particularly in studying protein aggregation, mitochondrial dysfunction, and oxidative stress. Despite the relative simplicity of yeast compared with human neurons, the conservation of key molecular pathways enables researchers to investigate fundamental aspects of neurodegeneration in a genetically tractable system.

Table 2 summarizes the neurodegenerative diseases discussed above, highlighting the yeast species used, the key proteins and mechanisms studied, as well as their applications, main advantages, and limitations.

Overall, the comparison presented in Table 2 highlights the versatility of yeast models in studying diverse neurodegenerative diseases while also emphasizing their shared limitations related to the lack of neuronal complexity. Expression of human disease-associated proteins in yeast enables high-throughput genetic and chemical screening, allowing systematic identification of conserved molecular modifiers and candidate therapeutic compounds. While yeast cannot fully replicate human brain pathology’s complexity, it is a crucial complementary tool alongside mammalian and patient-derived models.

Continued advancements in yeast-based research, combined with emerging technologies such as CRISPR genome editing and synthetic biology, will further enhance the utility of these models in neurodegenerative disease research. As the prevalence of these disorders continues to rise, leveraging yeast as a model system will remain vital in accelerating our understanding of disease progression and in the pursuit of novel therapeutic strategies.

### 4.4. Additional Disease Areas Informed by Yeast Models

#### 4.4.1. Yeast Prions as Models of Protein-Based Inheritance

Yeast prions provide a uniquely tractable system for dissecting the cellular principles of protein-based inheritance and amyloid biology. Classical yeast prions, such as PSI+, formed by the translation termination factor Sup35, and URE3, formed by the nitrogen catabolism regulator Ure2, represent self-templating amyloid conformers that can propagate heritable phenotypes without changes in the DNA sequence. Studies in *S. cerevisiae* have established concepts directly relevant to human prion and prion-like disorders, including conformational “strain-like” diversity and the distinction between aggregate formation and heritable transmission [98,99].

A major advantage of yeast prions is that their propagation can be analyzed as an emergent cellular phenotype shaped by chaperones, protein quality control systems, vesicular trafficking, and stress responses, enabling the mechanistic separation of amyloid nucleation, fragmentation, and transmission in living cells. Recent work further emphasizes that yeast encodes multiple anti-prion systems that suppress prion appearance and/or eliminate newly formed variants, providing a conceptual framework for endogenous anti-amyloid defenses in higher eukaryotes [99].

Cross-kingdom functional conservation has now been demonstrated experimentally: expression screens identified human proteins capable of curing yeast prions, indicating that conserved human proteostasis factors can directly antagonize prion states in vivo [100]. Together, these findings establish yeast prions as a foundational model for understanding both pathological and protective aspects of amyloid biology relevant to human neurodegenerative disease [98,99].

#### 4.4.2. Rare Neuromuscular Disease Proteinopathies (TDP-43)

Yeast has become a powerful platform for modeling rare neuromuscular and neurodegenerative diseases driven by protein misfolding and aggregation. A prominent example is TDP-43, whose aggregation and mislocalization underlie amyotrophic lateral sclerosis (ALS) and frontotemporal dementia (FTD). Expression of human TDP-43 or aggregation-prone fragments in yeast reproduces key disease-relevant features, including cytoplasmic mislocalization, aggregation, proteostasis overload (defined as saturation of the cellular protein quality control systems by misfolded proteins), and activation of conserved stress pathways, while facilitating the rapid discovery of genetic and chemical modifiers [101,102].

Because yeast supports scalable perturbation (deletions, overexpression, allele series), it is well-suited for rare-disease research, where patient numbers are limited, and many variants remain of uncertain significance. Yeast-based screens can identify conserved toxicity nodes (e.g., ER stress signaling, redox homeostasis, protein quality control) that can be prioritized for downstream validation in vertebrate and patient-derived models [101,102]. Recent work has shown that activation of the yeast cell-wall integrity MAPK pathway protects against TDP-43/TDP-25 toxicity, linking proteotoxic stress to adaptive stress signaling [103].

#### 4.4.3. Lipid Metabolism and Lysosomal Storage Disorders (Batten Disease)

Lysosomal storage disorders, linked to lipid metabolism, represent an additional disease class where yeast has provided mechanistic insights. Juvenile Batten disease is caused by mutations in *CLN3* and is increasingly viewed as a disorder integrating endolysosomal trafficking, lipid handling, and cellular stress responses. Yeast contributes through *BTN1*, the conserved ortholog of the human *CLN3* gene [104,105].

In yeast, *BTN1* deletion (btn1Δ) perturbs endosome-to-Golgi retrograde transport through altered SNARE assembly, providing a reductionist entry point into membrane-trafficking failures that are difficult to deconvolve in neurons [106]. Complementary mammalian studies suggest that *CLN3* influences additional processes, including stress granule dynamics and translational control [107]. Recent work in microglia links *CLN3* loss to impaired lipid metabolism and defective myelin turnover, highlighting an early immune-metabolic component of disease progression [105]. Together, the phenotypes of yeast btn1Δ mutants and mammalian data support a coherent lysosome–lipid–stress/inflammation axis in CLN3 disease biology [104,105,106,107].

#### 4.4.4. VPS13 Family Proteins and Lipid Transport at Contact Sites

The VPS13 family has emerged as a central component of intracellular lipid transport, functioning as bridge-like lipid conduits at membrane contact sites [108]. Mutations in VPS13A-D cause a spectrum of neurological disorders, and yeast has been instrumental in defining VPS13 function due to the ability to interrogate localization, contact-site dynamics, and trafficking phenotypes with high genetic precision [108,109].

Vps13 proteins constitute a conserved family of lipid transporters that function at multiple membrane contact sites, forming bridge-like channels to mediate lipid transfer between organelles and maintain lipid homeostasis [109,110,111]. In yeast, Vps13 localizes to several contact sites, including the ER–endosome interface, the vacuole–mitochondria patch, and the nucleus–vacuole junction (NVJ), highlighting its versatile role in lipid transport and organelle communication. These yeast studies provide a foundation for understanding VPS13 paralogs in vertebrates, where VPS13B and VPS13C display specialized functions at Golgi and ER–lysosome contact sites, respectively [110,111,112].

#### 4.4.5. Actin Cytoskeleton, Endocytosis, and Immunoactinopathies

Budding yeast was foundational in establishing endocytosis as an actin-driven, staged process in which actin polymerization supplies force and timing for membrane invagination and vesicle scission [113]. These discoveries have direct relevance to human disease, as mutations in actin regulators underlie immunoactinopathies that disrupt immune synapse function, migration, vesicular traffic, and receptor turnover [114].

Wiskott–Aldrich syndrome (WAS) is the archetypal immunodeficiency disorder: mutations in WASp impair Arp2/3-dependent actin remodeling, resulting in combined immunodeficiency with thrombocytopenia and immune dysregulation [115]. Although yeast lacks an immune system, the conserved actin machinery governing endocytosis provides a minimal mechanistic framework to understand how defective actin dynamics can secondarily impair trafficking-dependent signaling—an axis implicated across immunoactinopathies [113,114]. Yeast has also been used to model neuromuscular diseases. Mutations in GDAP1, causing Charcot–Marie–Tooth disease, and defects in myotubularins, linked to centronuclear myopathies, have been studied in yeast to explore mitochondrial function, lipid regulation, and organelle dynamics [116].

## 5. Application of Yeasts in Metabolic Disorders and Mitochondrial Diseases

### 5.1. Conventional and Unconventional Yeasts as Models in Metabolic Disorders and Mitochondrial Diseases

One of the trends observed in the development of modern biotechnology is the growing importance of specialized biological platforms that complement classic model organisms. Importantly, this change does not mean that classic model organisms are being replaced, as *S. cerevisiae* remains the dominant and well-recognized production host in many industrial and pharmaceutical applications. At the same time, unconventional yeasts, particularly *Y. lipolytica* and *P. pastoris*, have emerged as promising alternatives to traditional model yeasts such as *S. cerevisiae* for a range of biotechnological applications. Nevertheless, a systematic and comparative evaluation of their advantages and limitations relative to conventional yeasts remains necessary, especially in light of evolving industrial practices, regulatory frameworks, and the rapid development of synthetic biology tools [117].

When comparing conventional and unconventional yeasts, it becomes clear that their differences extend beyond individual metabolic pathways and influence overall biotechnological performance. *S. cerevisiae* is Crabtree-positive and preferentially ferments glucose even in the presence of oxygen, whereas *Y. lipolytica* and *P. pastoris* are Crabtree-negative and rely predominantly on respiratory metabolism. This distinction affects carbon flux distribution and product yield. However, under conditions of periodic cultivation with excess glucose, *P. pastoris* can partially suppress respiration and redirect carbon flow through alternative pathways, indicating a more complex metabolic regulation than a strictly respiratory phenotype [118,119].

A further key distinction lies in protein production. Unconventional yeasts have developed efficient protein secretion systems and are increasingly used for the production of recombinant proteins that are critical for vaccines and therapeutic applications. Species such as *P. pastoris* and *Y. lipolytica* are widely used due to their high secretion capacity and ability to perform post-translational modifications. They are also effective hosts for heterologous protein and biopharmaceutical production, providing valuable alternatives to traditional systems such as *S. cerevisiae* and *Escherichia coli* [120,121,122]. Altogether, these features define fundamental biological and technological differences between conventional and unconventional yeasts.

### 5.2. Mitochondria and Mitochondrial Diseases

In this context, mitochondria play an essential role in cellular function, controlling key processes such as energy production and the regulation of apoptosis. Consequently, assessment of mitochondrial function is central to studies of cellular metabolism [123]. Mitochondrial diseases (MDs) are inherited disorders caused by mitochondrial dysfunction arising from mutations in mitochondrial DNA (mtDNA) or nuclear genes encoding mitochondrial components [124]. These disorders affect approximately 1 in 5000 live births worldwide [125]. In recent years, MDs have attracted increasing attention from both researchers and clinicians, accompanied by improvements in diagnostic awareness and genetic counseling. However, effective treatments remain limited, and the molecular mechanisms underlying many MDs are still poorly understood [126].

Despite their heterogeneous genetic origins, MDs share a common link to impaired mitochondrial function. Although primary symptoms vary depending on the specific defect, clinical manifestations frequently converge within the broader spectrum of encephalomyopathy. Therefore, identifying defective cellular mechanisms is essential for understanding disease etiology and progression [127].

### 5.3. Yeast Models for Mitochondrial Genetics and Disease

Yeasts provide powerful experimental systems for dissecting mitochondrial biology, but individual species differ markedly in their suitability for specific research questions. These differences reflect variation in mitochondrial genetics, respiratory dependence, and the availability of molecular tools. Collectively, yeast models link tractable mitochondrial and nuclear genome manipulation with disease-relevant mitochondrial phenotypes, providing a robust framework for dissecting mechanisms and identifying therapeutic leads for human mitochondrial disorders (Table 3).

### 5.4. S. cerevisiae

*S. cerevisiae* enables the most advanced mitochondrial genetic manipulations and, uniquely among yeasts, supports direct transformation of mitochondrial DNA (mtDNA) via the ballistic (biolistic) method. In this approach, gold or tungsten particles coated with linear DNA are propelled at high velocity into intact cells, with delivery targeted to mitochondria. The introduced DNA integrates into mtDNA through highly efficient homologous recombination (HR; ~10^−4^ recombinants per cell), enabling precise engineering of point mutations in genes such as *COX1* or *ATP6* that recapitulate human pathogenic variants [128,129,130].

A major advantage of this system is homoplasmy selection, whereby all mitochondria within a cell harbor identical mtDNA. This allows rapid isolation of pure mutant lines under respiratory selection (e.g., growth on glycerol), so downstream phenotypes such as oxidative phosphorylation (OXPHOS) failure, elevated reactive oxygen species (ROS), or defects in mitochondrial biogenesis can be assessed within days rather than weeks. HR efficiency is further enhanced by high mtDNA copy number (50–100 copies per cell), recombination factors such as Mhr1 and Rad52, recombination hotspots, and mitochondrial fusion during cytoduction. Recombination outcomes are additionally shaped by cellular context, including stress conditions, growth phase, and intron content [131,132].

To quantify and validate HR and mitochondrial inheritance, studies commonly combine ARG8^m repeat assays, cytoduction crosses, and next-generation sequencing approaches that evaluate linkage disequilibrium decay or apply PHI-based recombination tests [133]. These tools support disease-oriented applications, as respiratory mutants can recapitulate features relevant to MELAS- and MERRF-like syndromes, petite screens have identified numerous factors involved in mitochondrial biogenesis, and lifespan–mtDNA interactions have been used to probe aging-related mechanisms. Respiratory-deficient petite (pet^−^) mutants, which are yeast colonies with defective mitochondria unable to grow on non-fermentable carbon sources, provide a practical system for assessing mitochondrial function [134,135]. Together, these capabilities establish *S. cerevisiae* as the gold standard for causal mtDNA variant testing, despite its lack of respiratory complex I and metabolic flexibility that necessitate careful growth conditions to unmask respiratory phenotypes [136,137].

### 5.5. S. pombe

*S. pombe* does not support direct mitochondrial transformation and instead relies on indirect HR-dependent strategies in a petite-negative background in which mtDNA is essential. As a result, research in this species emphasizes population-level analyses and mitochondrial dynamics rather than precise mtDNA engineering. Extensive natural mtDNA diversity documented across more than 192 isolates and dozens of mitogenome types enables modeling of heteroplasmy and analysis of clade-specific recombination using population sequencing approaches, including PHI tests and linkage disequilibrium decay [138,139].

In addition to this diversity-based framework, *S. pombe* exhibits moderate HR capacity through indirect routes such as nuclear–mitochondrial shuttling and petite reversion systems. The tight coupling of mitochondria to microtubules and the conservation of fission and fusion machinery provide clear, imageable readouts of mitochondrial division, segregation, and inheritance. Documented cases of biparental mitochondrial inheritance during meiosis further strengthen its value as a system for analyzing mtDNA transmission and organelle dynamics [140,141,142,143]. Overall, *S. pombe* prioritizes evolutionary diversity and inheritance dynamics over targeted mitochondrial genome manipulation.

### 5.6. Y. lipolytica

Although *Y. lipolytica* does not support direct mtDNA transformation and exhibits low-to-moderate levels of mitochondrial HR, respiratory-deficient petite (pet^−^) mutants and phenotype-driven approaches, such as growth on non-fermentable carbon sources, oxygen consumption measurements, and ATP production, provide robust functional readouts of oxidative phosphorylation. *Y. lipolytica* is uniquely suited for mitochondrial disease modeling because its mitochondrial architecture closely mirrors that of mammals at both genomic and enzymatic levels. Its circular ~47.9 kb mtDNA encodes key membrane subunits of the respiratory chain, including a complete, mammalian-like complex I. This complex comprises a nuclear-encoded hydrophilic arm, which contains the redox-active sites responsible for NADH oxidation and electron transfer, and an mtDNA-encoded membrane arm, embedded in the inner mitochondrial membrane and coupling electron transfer to proton translocation across the membrane. The conservation of complexes II–IV and ATP synthase further supports cross-complex analyses. In addition, the presence of an alternative oxidase provides a defined, cyanide-insensitive electron sink that modulates ROS production and metabolic flexibility, facilitating separation of primary OXPHOS defects from secondary redox effects. As an obligate aerobe with robust growth across a wide range of environmental conditions, *Y. lipolytica* displays pronounced phenotypes in response to mitochondrial dysfunction and supports applications linking mitochondrial activity to lipid metabolism. These features enable clear translation of molecular perturbations into disease-relevant phenotypes, including complex I-linked disorders such as Leigh syndrome, and position *Y. lipolytica* as the most promising translational yeast platform for modeling human mitochondrial diseases and evaluating therapeutic strategies [125,141,144,145,146,147,148,149,150,151] (Table 4). Table 5 summarizes a comparative evaluation of commonly used yeast models for MD research.

## 6. Cancer Research

### 6.1. Molecular Mechanisms Relevant to Cancer

#### 6.1.1. Chromosome Instability (CIN) and Discovery of Genes Associated with Cancer

Chromosome instability, encompassing aneuploidy, translocations, deletions, and structural rearrangements, is a hallmark of cancer cells and contributes to tumor heterogeneity and drug resistance [155,156]. Yeast genetic screening approaches, leveraging both non-essential gene deletions and temperature-sensitive essential gene alleles, have systematically uncovered genes whose loss promotes CIN phenotypes. Stirling et al. [157] screened approximately 2000 essential alleles in *S. cerevisiae*, identifying 692 CIN-associated genes. Many of these genes encode components of the mitotic spindle, kinetochore, replication machinery, and RNA processing complexes, functions similarly disrupted in human cancers. The high conservation of these pathways allows yeast CIN datasets to be mined for candidate cancer driver genes, providing a cost-effective preclinical filter before patient cohort validation [157].

#### 6.1.2. DNA Repair Pathways and Homologous Recombination

DNA double-strand breaks (DSBs) are among the most dangerous forms of DNA damage since they can cause chromosomal abnormalities and mutation accumulation if left unrepaired. This type of genomic instability is frequently observed in cancer cells and is considered a major driver of tumor progression [156]. Yeast has been a central model to dissect these repair mechanisms, particularly the homologous recombination (HR) pathway, which restores DNA by using an intact copy as a template. Studies in *S. cerevisiae* have been crucial for identifying the proteins involved in HR [158]. For example, Rad51 is the key recombinase that mediates DNA strand exchange, while Rad52 and Rad55 act as supporting factors to stabilize this process. In humans, the orthologs of these proteins, such as BRCA2 and BLM, are frequently mutated in hereditary cancer syndromes, leading to defective DNA repair and increased cancer susceptibility [158]. Because of this conservation, yeast systems provide an effective tool for testing the functional impact of cancer-related mutations and for classifying pathogenic variants. Moreover, the mechanistic insights from yeast studies have directly influenced cancer therapy. For instance, PARP inhibitors, now widely used in BRCA-mutated tumors, exploit the vulnerabilities in DNA repair pathways first characterized in yeast [159].

#### 6.1.3. Telomere Dynamics and Genomic Stability

Telomeres are protective structures that prevent degradation and abnormal repair of chromosome ends. Under normal conditions, telomeres shorten progressively with each cell division; once they reach a critical length, cells undergo senescence or apoptosis. Cancer cells, however, bypass this natural limit by reactivating telomerase or by engaging alternative lengthening of telomeres (ALT) mechanisms [160].

Yeast provides a powerful model to investigate how telomere length is regulated. Studies in *S. cerevisiae* have shown that telomere maintenance depends on telomerase, telomere-binding protein complexes, and upstream regulatory assemblies such as the ASTRA and TTT complexes. The loss of TTT components (Tti1 or Asa1) reduces cellular levels of Tel1p, the yeast homolog of human ATM (ataxia-telangiectasia mutated) kinase, which is a central regulator of DNA damage signaling and telomere length control. Reduced Tel1p/ATM activity leads to telomere shortening, highlighting the strong evolutionary conservation of telomere regulation mechanisms between yeast and humans [157]. As such, yeast models provide a genetically tractable platform for dissecting telomerase activity, telomere-capping mechanisms, and the ALT pathways frequently activated in cancer cells.

### 6.2. Yeast as a Functional Model in Cancer Research

#### 6.2.1. Yeast Models for Drug Sensitivity and Resistance

One of the most powerful applications of yeast in cancer research is their use as surrogate models to study drug–gene interactions. Because many signaling and metabolic pathways are conserved, anticancer agents can be tested in yeast to reveal their mechanism of action and identify potential resistance mechanisms. For example, DNA-damaging drugs such as cisplatin induce a strong checkpoint response in *S. cerevisiae*, allowing researchers to analyze the role of repair proteins in conferring resistance [161]. Yeast “chemogenomic profiling,” in which thousands of deletion strains are exposed to a drug, can reveal the full network of genes influencing drug sensitivity. This has been successfully used to predict resistance pathways in human tumors, highlighting the translational power of yeast in preclinical screening. Furthermore, heterologous expression of human genes associated with cancer in yeast (such as *BRCA1* or oncogenic kinases) allows direct testing of how specific drugs modulate these targets [162]. Yeast-based assays thus provide a high-throughput, cost-effective model for probing drug–target interactions long before clinical testing in mammalian cancer cells.

#### 6.2.2. Functional Studies of Human Genes Associated with Cancer and Modeling Genomic Instability

While Section 6.1.1 focuses on the use of yeast to identify genes associated with chromosome instability, this section highlights how yeast models are used to functionally dissect the cellular consequences of genomic instability and cancer-associated mutations.

Yeast represents a valuable system for the functional validation of human genes associated with cancer and for modeling the cellular consequences of genome instability, a central hallmark of cancer. Because yeast cells tolerate heterologous protein expression, researchers can introduce human genes associated with cancer, such as BRCA1 and BRCA2, to study their functions in a simplified and genetically tractable setting [22]. These genes have been expressed in *S. cerevisiae* to investigate conserved aspects of homologous recombination, including the direct interaction of BRCA2 with the recombinase Rad51 and the regulatory role of BRCA1 in DNA damage response pathways. Such studies have provided critical mechanistic insights into DNA repair processes that are frequently disrupted in human tumors.

Mutational analysis in yeast further enables rapid functional screening of cancer-associated variants and helps classify whether specific mutations are pathogenic or benign [163]. This approach is particularly useful for interpreting variants of uncertain significance identified in large-scale cancer sequencing studies.

Functional modeling in yeast extends beyond DNA repair pathways. Genes involved in cell cycle regulation, particularly cyclin-dependent kinases (CDKs) and their regulators, can be effectively studied in *Schizosaccharomyces pombe* [164]. With its G2-dominant cell cycle that closely mirrors that of human cells, *S. pombe* has proven invaluable for dissecting mitotic errors, chromosome segregation defects, and checkpoint failures—processes that are central to uncontrolled cancer cell proliferation [165].

Yeast also provides a powerful platform for modeling the cellular consequences of aneuploidy. Experimentally generated aneuploid yeast strains exhibit metabolic stress, proteotoxic imbalance, and reduced growth fitness, phenotypes that closely resemble those observed in human cancer cells with abnormal karyotypes [166]. These conserved stress responses allow yeast to serve as a cost-effective experimental filter for prioritizing candidate driver genes and pathways emerging from large-scale cancer genomics projects.

To facilitate comparison of the genetic tractability of yeast models discussed in the previous section, Table 6 summarizes the genetic tools, biological processes, and cancer-related applications described for *S. cerevisiae* and *S. pombe*.

**Figure 1:**
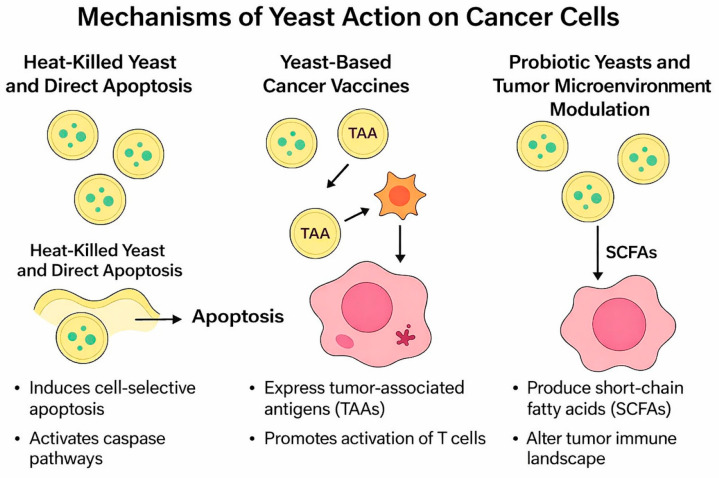
Mechanisms of yeast action on cancer cells.

Figure 1 illustrates three different mechanisms by which yeasts affect cancer cells. On the left, heat-killed yeast cells are shown inducing apoptosis, highlighting their ability to selectively trigger programmed cell death through caspase activation. In the middle, recombinant *S. cerevisiae* expressing tumor-associated antigens (TAAs) is depicted, demonstrating how yeast-based vaccines activate T-cell responses against tumor cells. On the right, probiotic yeasts such as *S. boulardii* are represented, producing short-chain fatty acids (SCFAs), which reshape the tumor microenvironment, reduce inflammation, and enhance immune surveillance.

#### 6.2.3. Synthetic Lethality Screens

One of the most transformative contributions of yeast to cancer biology has been the development of synthetic lethality (SL) screening platforms. Synthetic lethality occurs when the simultaneous disruption of two genes leads to cell death, whereas alteration of either gene alone is tolerated. In oncology, this principle provides an elegant therapeutic strategy: drugs can be designed to target a gene that is only essential in the presence of a cancer-specific mutation, thereby sparing normal cells [20].

Yeast is particularly powerful for SL discovery because of its unparalleled genetic tractability. With large-scale deletion collections, temperature-sensitive alleles, and CRISPR-based perturbation systems, researchers can systematically test millions of gene–gene combinations. High-throughput screening platforms, such as synthetic genetic array (SGA) analysis, have enabled the construction of comprehensive genetic interaction networks in *S. cerevisiae* [20]. These maps reveal both buffering interactions that maintain cellular robustness and lethal pairs that uncover hidden vulnerabilities. Many of these interactions have conserved counterparts in mammalian cells, underscoring the translational relevance of yeast findings [167].

From a cancer research perspective, yeast SL studies serve two major roles. First, they provide mechanistic insights into pathways frequently dysregulated in tumors, such as DNA repair, chromosome segregation, and cell-cycle checkpoints. For example, interactions between homologous recombination factors and DNA replication proteins identified in yeast have parallels in BRCA-deficient cancers [158]. Second, yeast offers a rapid preclinical filter to prioritize therapeutic targets. The identification of conserved SL interactions has directly inspired targeted therapies, such as PARP inhibitors used to exploit the vulnerability of BRCA-mutant tumors [159].

Recent advances further enhance the utility of yeast SL models. Integration of chemical–genetic interaction screens allows testing of small molecules in combination with genetic perturbations, directly mimicking drug treatment scenarios in cancer cells [168]. Additionally, cross-species comparative approaches now enable validation of SL pairs in yeast, human cell lines, and even patient-derived tumor organoids, creating a powerful translational pipeline. Synthetic lethality screens in yeast represent a unique intersection of fundamental genetics and translational oncology.

### 6.3. Translational and Clinical Applications of Yeast in Cancer

Yeast has moved beyond its role as a model organism to become a versatile tool with direct applications in translational oncology [169]. Through advances in genetic engineering, metabolic rewiring, and immunological exploitation, yeast contributes to multiple aspects of cancer therapy, from drug production to vaccine development and microbiome-based interventions [170].

One of the most prominent applications lies in the bioproduction of anticancer compounds and therapeutic antibodies. Non-conventional yeasts such as *P. pastoris* have been produced in engineered plant-derived alkaloids like noscapine and paclitaxel, two clinically important molecules traditionally obtained from natural sources at low yield [170]. Moreover, glycoengineering efforts have enabled *P. pastoris* to produce humanized monoclonal antibodies with improved therapeutic efficacy. For example, HER2-targeted antibodies such as trastuzumab have been successfully expressed in engineered yeast, showing glycosylation and performance comparable to mammalian cell-derived products [171]. Compared with Chinese Hamster Ovary (CHO) cells, the current industry standard yeast offers faster growth, lower production costs, and scalability, making it a complementary platform for rapid and cost-effective drug manufacturing [172]. With the integration of CRISPR-based genome editing and synthetic biology tools, yeast biofactories now hold promise for producing next-generation biologics with high precision [36].

Yeast also provides innovative opportunities in cancer immunotherapy and supportive care. Recombinant *S. cerevisiae* strains expressing tumor-associated antigens (TAAs) have been developed as therapeutic cancer vaccines [173]. Once administered, these engineered yeasts are taken up by antigen-presenting cells and trigger antigen-specific T-cell responses, supported by the natural adjuvant effects of β-glucans present in the yeast cell wall. Preclinical studies demonstrated that yeast-based vaccines could induce cytotoxic T lymphocytes capable of killing tumor cells in colorectal, pancreatic, and thyroid cancer models [173,174].

At the same time, probiotic yeasts such as *S. boulardii* play a supportive role in cancer therapy by modulating the gut microbiome, strengthening the intestinal barrier, and producing short-chain fatty acids (SCFAs) with direct anti-proliferative and immunomodulatory effects [175]. These properties are especially relevant for patients undergoing chemotherapy or radiotherapy, where microbiome imbalance and inflammation contribute to disease progression and therapy-related complications [170].

Taken together, yeast-based systems represent a unique bridge between fundamental cancer biology and translational medicine. Whether as biomanufacturing platforms for anticancer molecules, immunotherapeutic delivery systems, or probiotic modulators of the tumor microenvironment, yeasts provide scalable, low-cost, and versatile strategies that complement conventional cancer treatments while opening new frontiers in precision oncology.

## 7. Drug Discovery

Yeasts have become essential in high-throughput screening (HTS), allowing researchers to test thousands of compounds for their biological effects. Several yeast-based approaches facilitate drug target identification, including deletion libraries, chemical–genetic interactions, yeast two-hybrid screening, dual-bait system use, and CRISPR-based functional genomics. These techniques help identify potential therapeutic targets and rapidly uncover mechanisms of drug action at an unprecedented scale [176,177,178].

To provide an overview of the main yeast-based strategies used in drug discovery, Table 7 summarizes the main screening strategies, their core principles, and major applications.

There are five model yeast organisms most often used in drug discovery. *S. cerevisiae* is a primary model for HTS, chemical genomics (screening of targeted chemical libraries of small molecules against individual drug target families), protein–protein interactions, and modeling conserved pathways (cancer, neurodegeneration or G protein-coupled receptors—GPCRs) [179,180,181]. *S. pombe* is used as a model eukaryote for HTS [182,183]. *C. albicans* and *C. glabrata* are studied directly to understand antifungal resistance and multidrug resistance (MDR) mechanisms, due to their importance as human pathogens [184,185]. At last, *P. pastoris* is used as a microbial cell factory for the efficient production of biopharmaceuticals [186,187].

**Figure 2:**
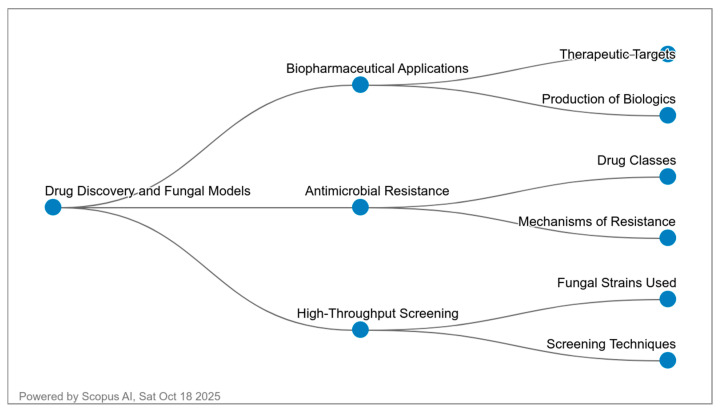
Concept map automatically generated online using Scopus AI (Elsevier, Amsterdam, The Netherlands) https://www.elsevier.com/en-au/products/scopus/scopus-ai accessed on 18 October 2025, illustrating research topics related to yeast models in drug production.

The diagram in Figure 2 illustrates three main research areas involving yeasts in pharmacology. The central node, Drug Discovery and Fungal Models, connects to (1) Biopharmaceutical Applications, representing the use of yeasts such as *P. pastoris* and *Y. lipolytica* as microbial factories for biologics and therapeutic proteins; (2) Antimicrobial Resistance, highlighting studies on *C. albicans* and *C. glabrata* to understand drug classes and resistance mechanisms; and (3) High-Throughput Screening, where model yeasts like *S. cerevisiae* and *S. pombe* are applied for large-scale chemical screening and pathway modeling. Together, these branches illustrate how yeast systems contribute to modern drug discovery, production, and the analysis of drug resistance.

Genomic engineering of yeast, particularly the species *S. cerevisiae*, is an extremely valuable tool that facilitates the discovery of drugs for human diseases due to the yeast’s high susceptibility to genetic manipulation, rapid growth, and the conservation of cellular pathways compared to human cells [179,188,189]. Yeast serves as an advanced screening platform, enabling the identification of new drug targets, determination of compound mechanisms of action (MOA), and modeling of specific human diseases, such as cancer and neurodegenerative disorders [84,180]. Key ways in which yeast genomic engineering aids drug discovery include: chemogenomic profiling and target identification, modeling specific human diseases, and targeting G Protein-Coupled Receptors (GPCRs).

Genomic engineering has allowed for the creation of complete yeast strain collections with precise gene deletions. Deletion libraries are collections of yeast strains, each missing a single non-essential gene, which allows systematic screening to identify genes involved in drug sensitivity or resistance. The Yeast KnockOut (YKO) collection consists of a complete set of deletion strains, including haploid strains of both mating types, as well as heterozygous and homozygous diploid deletions. These strains are tagged with unique 20-base pair sequences, or “barcodes,” allowing them to be pooled and screened competitively in bulk [190,191].

Chemical–genetic interactions can be investigated by combining small molecules with yeast mutants. Researchers can map pathways affected by drugs and identify novel drug targets. Drug-induced Haploinsufficiency Profiling (HIP) and Homozygous Profiling (HOP) are chemical–genetic screening methods used in *S. cerevisiae* to identify drug targets and understand drug mechanisms. HIP utilizes the heterozygous diploid deletion strains [175]. If a small molecule targets the product of an essential gene, the corresponding heterozygous strain becomes disproportionately sensitive to the drug. HIP allows the simultaneous identification of drug target candidates and inhibitory compounds in vivo, confirming the mode of action for known drugs, such as methotrexate [176]. HOP (or haploid deletion profiling) uses strains lacking a complete, non-essential gene. HOP identifies genes and pathways that buffer the drug target pathway or are involved in resistance mechanisms [20,176]. Complementary to HIP, Multi-Copy Suppression Profiling (MSP) identifies genes that, when overexpressed, confer resistance to the inhibitory compound, e.g., successfully identifying the target gene of rapamycin [176,192]. Engineered yeast strains in High-Throughput Morphological Profiling (HTP), such as the strain lacking drug efflux pump genes, are used to measure dose-induced morphological changes. HTP allows for comparing these changes to gene deletion signatures, allowing for the objective prediction of intracellular targets, aiding target deconvolution. For instance, this method helped identify the proteasome regulatory particle as the target of bortezomib [178].

The yeast two-hybrid (Y2H) screening technique identifies protein–protein interactions, which are crucial for studying drug targets that function as part of multi-protein complexes. The Y2H system is the gold standard method for mapping and characterizing novel protein–protein interactions (PPIs) [189,193]. The principle relies on reconstituting a functional transcription factor through the physical interaction of fused “bait” (protein of interest fused to the DNA-binding domain (DBD) of a transcription factor) and “prey” (a second protein fused to the activation domain (ADo) of a transcription factor) proteins, activating a reporter gene. If the two proteins (bait and prey) physically interact, their association brings the DBD and the ADo into close proximity, reconstituting a functional hybrid transcription factor. This reconstitution activates the transcription of a downstream reporter gene [189]. As an example, the Y2H system has been critical in characterizing the interactome of Protein Phosphatase 1 (PP1) isoforms in human testis, revealing key regulatory subunits and highlighting these complexes as therapeutic targets [194,195,196,197].

Dual-bait systems (or variants thereof, such as the membrane-based split-ubiquitin system) extend classical yeast two-hybrid approaches by enabling the analysis of protein–protein interactions (PPIs) involving membrane-associated proteins that cannot be transported to the nucleus [189,198,199,200]. In this system, the yeast cell is engineered to contain two distinct “bait” proteins and a “prey” protein, with each component linked to separate reporter genes [200]. The Reverse Yeast Two-Hybrid (rY2H) system identifies small molecules that disrupt therapeutically relevant PPIs [189]. In this system, interaction between proteins activates a toxic gene, leading to cell death, whereas cell survival indicates successful disruption of the PPI by the small molecule [189]. This method is particularly crucial and valuable because it inherently screens for membrane-permeable and non-toxic compounds [189,201].

The Yeast Three-Hybrid (Y3H) system is designed to detect interactions between a protein and a small molecule drug [202]. This is crucial for profiling small molecules to discover their entire spectrum of targets [189]. The small molecule is chemically coupled to an anchor compound to display the ligand inside the cell. Any interacting prey protein activates the reporter gene [202,203]. Studies confirmed that the level of transcription activation in Y3H correlates with the strength of the ligand–receptor binding affinity, demonstrating the requisite sensitivity (e.g., cutoff around 50 nM) for drug discovery applications [203].

CRISPR-Cas9 technology has been adapted for yeast to enable gene editing and high-throughput screening of gene function in response to drug treatment, such as the Quasi-WT reference strain used in GPCR studies [204]. *S. cerevisiae* naturally utilizes a pheromone signaling pathway homologous to mammalian GPCR pathways [205,206]. Heterologous human GPCRs can be functionally coupled to this pathway to create screening assays [205]. Genome engineering has created modular GPCR signal transduction systems, allowing predictable tuning of the cellular response [205]. Advanced methods like the split-ubiquitin system combined with G-protein signaling assays enable simultaneous monitoring of GPCR dimerization and ligand-mediated signaling [207,208].

**Table 7:** Yeast-based strategies used in drug discovery.

Screening Strategy	Core Principle	Yeast Species Used	Main Application	References
High-throughput phenotypic screening	Growth- or phenotype-based screening of compound libraries	*S. cerevisiae*, *S. pombe*	Identification of bioactive compounds and modifiers of toxicity	[176,177,178,179,180,181,182,183]
Chemogenomic profiling	Analysis of drug–gene interactions using mutant collections	*S. cerevisiae*	Identification of mechanisms of action and drug targets	[176,190,191]
Haploinsufficiency profiling	Increased drug sensitivity of heterozygous deletion strains	*S. cerevisiae*	Identification of direct molecular targets	[176,190,191]
Homozygous profiling	Drug sensitivity of non-essential gene deletions	*S. cerevisiae*	Identification of buffering and resistance pathways	[20,176]
Multicopy suppression profiling	Suppression of toxicity by gene overexpression	*S. cerevisiae*	Identification of compensatory pathways and drug targets	[176,192]
Morphological profiling	Drug-induced cellular morphology changes	*S. cerevisiae*	Prediction of intracellular targets based on phenotypic similarity	[178]
Protein–protein interaction screening	Detection of protein–protein interactions and identification of interaction modulators	*S. cerevisiae*	Identification of druggable protein complexes	[189,193,194,195,196,197]
Dual-bait/reverse Y2H systems	Selection based on disruption of protein–protein interactions	*S. cerevisiae*	Screening of small molecules interfering with PPIs	[189,198,199,200,201]
Yeast three-hybrid (Y3H)	Detection of ligand–protein interactions	*S. cerevisiae*	Identification of direct molecular targets of small molecules	[202,203]
GPCR-based screening	Coupling of human GPCRs to yeast signaling pathways	*S. cerevisiae*	Functional screening and characterization of GPCR ligands	[204,205,206]
CRISPR-based screening	Targeted genome perturbation and functional validation	*S. cerevisiae*	Functional validation of candidate drug targets	[204,205]
Antifungal resistance screening	Analysis of multidrug resistance and efflux mechanisms	*C. albicans*, *C. glabrata*	Identification of antifungal resistance mechanisms	[184,185]
Biotechnological/production-oriented screening	Selection of strains with improved expression capacity	*P. pastoris*	Production of therapeutic proteins and biologics	[162,171,172,186,187]

Abbreviations: CRISPR—clustered regularly interspaced short palindromic repeats; Y2H—yeast two-hybrid; Y3H—yeast three-hybrid; PPIs—protein–protein interactions; GPCRs—G protein-coupled receptors.

Yeast Surface Display (YSD) technology, combined with Phage Display, allows for the rapid screening of therapeutic peptides targeting aggregation-prone proteins associated with neurodegenerative disorders [209].

Despite their many advantages, yeast models have some limitations in drug discovery. Yeast cells lack human-like complexity, including certain organelles, immune responses, and signaling pathways present in mammalian systems. While yeast can reveal fundamental insights, additional validation in mammalian cells or animal models is often necessary because yeast are unable to model complex tissue interactions or immune responses [31,177,179].

Yeasts metabolize drugs differently from human cells, which can affect drug screening results. Co-expression of human drug-metabolizing enzymes in yeast is one approach to address this limitation [210]. Still, the rigid cell wall restricts compound entry, complicating translation to mammalian systems [179]. Yeast cells possess a pleiotropic drug resistance (PDR) network [211], with efflux pumps (Pdr5p, Snq2p) actively expelling small molecules [212]. Those efflux pumps are involved in extruding a wide variety of toxic compounds, such as drugs, alkanes, and caffeine, from the cell. These ATP-binding cassette transporters contribute to cellular multidrug resistance by preventing the accumulation of toxic substances to lethal levels. They are often regulated by transcription factors like Pdr1p and Pdr3p. This leads to false negative screening results unless engineered strains lacking these pumps are used [176,178]. Furthermore, the Yeast Three-Hybrid (Y3H) system requires chemical coupling of the drug to an anchor, which can alter the drug’s inherent properties [203].

Advances in synthetic biology, genome editing, and machine learning are expanding yeast’s applications in drug discovery. Future directions include engineering yeasts with humanized pathways, improving yeast-based high-throughput screening (HTS) platforms, and integrating yeast models with other systems such as organoids and AI-driven drug design [213]. To conclude, there are already many solutions that can help circumvent some of these limitations, and awareness of their existence allows for correct reasoning.

## 8. Limitations and Challenges of the Yeast Model

The application of yeasts as a model for human diseases comes with some limitations and challenges. Yeast is a unicellular organism that engages directly with its environment. This contrasts with the cells of multicellular organisms, which are partially insulated through strict homeostasis [214]. Due to their unicellular nature, *S. cerevisiae* and other yeasts lack the intricate organ systems present in multicellular organisms, limiting their applicability in research focused on tissue differentiation, organ function, and overall physiology. Gershon & Gershon [212] paid attention to *S. cerevisiae* as a model for aging research. However, its unicellular nature limits its ability to model the complex, system-wide processes involved in aging in multicellular organisms. Yeast lacks the cellular specialization and intercellular communication seen in multicellular organisms, making it unable to model aging processes that depend on these features. Moreover, while yeast cell dysfunction has little impact on the cell community, the loss of specialized cells (e.g., neurons or muscle cells) in multicellular organisms can severely impair tissue or organ function. Additionally, yeast cannot replicate the systemic homeostasis and coordinated stress responses (e.g., hypoxia adaptation via HIF-1) that are critical in multicellular organisms [214]. Relatedly, some human processes emerge only from tissue-level context (e.g., endocrine signaling, immune crosstalk, vascularization), which cannot be recapitulated in a single-celled model. These constraints motivate careful selection of questions where cell-autonomous mechanisms dominate, reserving multicellular models for higher-order physiology.

Yeast does not have an adaptive immune system or the specialized immune cells (e.g., T cells and B cells) found in higher eukaryotes. Thus, it is unsuitable for studying immune responses, inflammation, or host–pathogen interactions relevant to human diseases. However, yeast can be used to study basic innate immune mechanisms, such as autophagy [215] and stress responses [216], but these processes are often more complex in multicellular organisms. More frequently, yeasts can be utilized to explore fundamental aspects of innate immunity. For instance, cell wall extracts from *S. cerevisiae* with varied α-mannan and β-1,3-glucans chain lengths influenced the innate immunity and mucosal tissue responses of the intestine of zebrafish (*Danio rerio*) [217].

Only 31% to almost 50% of yeast genes have human orthologs, limiting the modeling of many human-specific processes [188,189]. Accordingly, yeast excels as a reductionist system to reconstitute single pathways (e.g., autophagy modules) or to produce defined microbe-associated molecular patterns that stimulate vertebrate models, rather than to model whole-organism immunity. Compared to mammals, yeasts also exhibit significant metabolic differences, which can limit their applicability in various research areas, including energy production, nutrient utilization, drug metabolism, and secondary metabolite biosynthesis. The availability of nutrients is essential for cell survival. The TOR (target of rapamycin) pathway is instrumental in regulating cell growth based on nutrient levels, particularly amino acids. Additionally, mTOR (mammalian TOR) is linked to various diseases such as cancer, obesity, and diabetes. Interestingly, recently identified mammalian cytosolic sensors for leucine and arginine do not have direct equivalents in yeast, although they functionally interact with GATOR2 (Gap Activity Towards Rags), which has a yeast counterpart known as SEACAT. González and Hall [218] noted that the reasons for this discrepancy remain unclear, indicating that further research is needed to investigate how amino acids might affect SEACAT in yeast. Beyond signaling, differences in lipid composition (e.g., ergosterol in yeast versus cholesterol in mammals), organelle architecture, and cofactor availability can alter how human enzymes behave when transplanted into yeast, occasionally necessitating co-expression of human chaperones or lipid-modifying enzymes.

Cell growth is vital for organismal development because it allows organisms to increase in size, which is as important as cell division. Conlon & Raff’s study [219] using primary rat Schwann cells and yeasts revealed that cells exhibit fundamentally different lifestyles. Yeasts are single-celled organisms that rapidly grow and divide depending on nutrient levels, requiring them to swiftly adapt to shifting environments. Conversely, the growth and division of animal cells are meticulously controlled through intercellular signaling, which advantages the whole organism. Consequently, even small fluctuations in these signals can significantly impact cell growth and proliferation. This divergence means that drug responses or toxicity observed in yeast may reflect cell-autonomous effects but not organism-level regulation, reinforcing the need for orthogonal validation.

Researchers can implement various strategies to tackle the challenges posed by yeast as a model organism. In addition to the use of advanced methods of genetic engineering, genome editing using the CRISPR system, and other genome editing methods, the use of systems biology seems to be equally important. Systems biology marks a transition from focusing on single genes or proteins to examining complete pathways, cellular processes, and organisms’ systems [220]. Yeast has been crucial in advancing systems biology, functioning as a model organism and a cell factory. Its experimental versatility allows for precise manipulation of growth conditions and nutrient composition, enabling the generation of high-resolution multi-omics data [221]. This has provided valuable insights into cellular physiology, including mechanisms related to complex human diseases such as cancer, diabetes, and metabolic disorders [222]. According to Nielsen [9], systems biology utilizes computational and mathematical modeling to investigate these interactions through two primary approaches. The first one is called top-down systems biology, which focuses on omics data and network models, and the second is bottom-up systems biology, which develops mathematical models based on biological knowledge of specific pathways or subsystems [9]. Significant progress in yeast systems biology has come from incorporating big data technologies to investigate gene expression dynamics, cellular metabolism, and the regulatory networks that connect these processes. The development of genome-scale metabolic models and the application of machine-learning techniques have greatly improved predictive capabilities [9,223]. By integrating experimental control, multi-omics data, and computational modeling, yeast systems biology effectively bridges the divide between fundamental research and practical applications in biotechnology and medicine. Complementary tactics to increase model fidelity include: (i) pathway-level humanization (swapping multiple enzymes or entire complexes), (ii) expression tuning to approximate human dosage, and (iii) “environmental humanization” (media and lipid remodeling) to align biophysical context. Finally, standardized reporting of constructs, strain backgrounds, and phenotype metrics will enhance reproducibility and facilitate meta-analyses across studies.

Importantly, many limitations inherent to yeast models can be addressed through complementary experimental systems rather than treated as prohibitive constraints. While yeast is excellent for dissecting conserved, cell-autonomous mechanisms, higher-order models such as mammalian cell cultures, organoids, and co-culture systems are better suited to capture tissue-specific regulation, multicellular organization, and cell–cell interactions [224]. For instance, hypotheses and candidate modifiers identified in yeast can be validated in neuronal or cancer organoids, while co-culture systems incorporating immune or stromal cells allow investigation of signaling contexts that are absent in unicellular models [225]. In this integrated approach, yeast serves as an efficient platform for discovery and prioritization within multi-model translational research pipelines, rather than as a stand-alone surrogate for complex human physiology [226].

## 9. Future Directions and Conclusions

Over recent decades, yeast has transitioned from a traditional genetic model to a flexible system for exploring human disease mechanisms, discovering therapeutic targets, and producing relevant biomolecules. This review’s examples, ranging from neurodegeneration and mitochondrial disorders to cancer and drug discovery, demonstrate how conserved eukaryotic processes can be leveraged in a simple, manageable organism to generate insights that are often challenging in more complex systems.

Looking forward, one promising area is the ongoing “deep humanization” of yeast. Early efforts involved expressing individual human proteins or substituting single yeast genes with their human equivalents. Recent innovations now enable the systematic replacement of entire gene sets and pathways, such as glycolysis, DNA repair, and mitochondrial functions. Extending these methods to multi-gene pathways, complex protein assemblies, and regulatory networks will allow for more realistic modeling of human cell biology in yeast. When combined with large-scale testing of patient-derived variants, these platforms will facilitate more accurate variant classification and research into disease mechanisms.

Another key frontier is the closer integration of yeast genetics with multi-omics and systems biology. Genome-wide perturbation tools like CRISPR editing, barcoded deletion, and overexpression libraries—paired with transcriptomic, proteomic, metabolomic, and lipidomic profiling—will enable large-scale mapping of how disease mutations reprogram cellular networks. Incorporating computational modeling and machine learning can predict emergent behaviors, help prioritize genetic interactions for validation, and guide the design of synthetic circuits or engineered strains. Such approaches will be especially valuable for complex traits such as drug resistance, metabolic reprogramming in cancer, or compensatory pathways in mitochondrial issues.

Third, yeast is set to become an even more vital preliminary screening tool in translational research. High-throughput genetic and chemical screens can quickly identify potential disease modifiers, synthetic lethal partners, and small molecules targeting conserved pathways. Although validation in mammalian cells, organoids, and in vivo models remains essential for clinical relevance, initial discovery and screening in yeast offer a cost-effective and rapid alternative, especially beneficial in precision oncology, where humanized yeast strains expressing tumor variants or rewired signals can help prioritize drug combinations or uncover vulnerabilities linked to specific mutations.

Simultaneously, non-conventional yeasts will expand the range of diseases that can be modeled. Species such as *Y. lipolytica* and *S. pombe* offer unique advantages for studying mitochondrial biology, cell cycle regulation, and organelle behavior. *P. pastoris* and other production strains serve as efficient biofactories for complex biologics and vaccines. Rationally selecting and combining these models, rather than relying solely on *S. cerevisiae*, will enhance pathway coverage and enable more detailed modeling of mitochondrial diseases, degenerative disorders, and therapies.

Despite these advances, several significant challenges remain. Key unresolved biological questions include which human disease phenotypes are truly cell-autonomous and therefore suitable for yeast modeling, how multi-gene and pathway-level defects can be accurately reconstructed, and to what extent compensatory mechanisms in yeast hide disease-relevant vulnerabilities. From a technical standpoint, major obstacles include incomplete conservation of certain human pathways, differences in membrane composition and post-translational modifications, limitations in modeling tissue-specific regulation, and the need to move beyond single-gene replacement toward pathway-level humanization. In translational settings, regulatory and practical concerns also arise, particularly regarding the standardization and reproducibility of humanized yeast platforms, integration of yeast-derived data with mammalian validation pipelines, and acceptance of yeast-based findings as solid preclinical evidence in drug development. Overcoming these challenges will be crucial for fully integrating yeast into multi-model translational research strategies.

Despite these limitations, understanding yeast’s constraints remains crucial. Yeast cannot replicate tissue structure, multicellular homeostasis, endocrine, or immune signaling, nor capture the full brain complexity. Many human genes have no clear yeast equivalents, and differences in metabolism, membrane makeup, and organelle architecture influence how human proteins behave in yeast. Going forward, yeast will increasingly serve as one component of multi-model strategies: a simplified system for analyzing cell-intrinsic mechanisms, screening extensive variant or compound libraries, and generating hypotheses that are subsequently tested in higher complexity models.

Progress will also rely on community standards and tool development. Accessible strain collections, humanized gene panels, barcoded libraries, and standardized assays will promote reproducibility and facilitate meta-analyses across laboratories. Open data formats and computational pipelines are essential for integrating yeast data with clinical genomics, patient registries, and drug databases. Embracing FAIR (Findable, Accessible, Interoperable, Reusable) principles will further ensure that knowledge derived from yeast is readily applicable in translational research.

In summary, yeast has become a key platform for exploring the molecular mechanisms of human diseases and advancing therapeutic development. Its distinct advantages, genetic accessibility, evolutionary conservation, and compatibility with high-throughput methods make it an essential complement to mammalian and patient-derived models. By integrating deep humanization, systems biology, synthetic biology, and translational screening, future studies will continue to harness yeast’s potential to connect fundamental molecular insights with clinical applications.

## Figures and Tables

**Table 1:** Key Advantages of Yeast Models for Infectious Disease Research.

Aspect	Benefit in Infectious Disease Research	Example Application
Genetic Tools	High-throughput mutagenesis and CRISPR editing	Viral variant evolution [47]
Conserved Pathways	Mimics eukaryotic host responses without complexity	Vesicle trafficking disruption [49]
Scalability	Cost-effective screening of protein functions	RBD affinity assays [50]
Non-Mammalian	Avoids ethical issues in early-stage pathogen studies	Immune modulator testing [51]

Abbreviations: RBD—receptor-binding domain.

**Table 2:** Yeast models in neurodegenerative diseases.

Neurodegenerative Disease	Yeast Species Used As Models	Studied Proteins/ Mechanisms	Applications of Yeast Models	Advantages	Limitations	References
Alzheimer’s disease (AD)	*S. cerevisiae*, *S. pombe*, *C. glabrata*, *K. lactis*, *Y. lipolytica*	Aβ (Aβ40, Aβ42, Aβ43), APP, presenilin-1, presenilin-2, Tau; protein aggregation, oxidative stress, mitochondrial dysfunction	Analysis of Aβ and Tau aggregation; secretase activity; modeling toxicity; identification of aggregation modifiers, autophagy studies	Conserved cellular pathways; heterologous expression of human proteins	Lack of neurons and endogenous APP processing; simplified pathology	[52,53,54,55,56,57,58,59,60,61,62,63,64,65,66,67,68,69,70]
Parkinson’s disease (PD)	*S. cerevisiae*, *S. pombe*	α-synuclein aggregation, vesicle transport, proteostasis, oxidative stress	Modeling α-synuclein toxicity; identification of genetic modifiers; screening of protective compounds	Reproducible α-synuclein toxicity; suitability for compound screening	Absence of dopaminergic neurons and motor phenotypes	[66,71,72,73,74,75,76,77,78,79,80,81,82]
Huntington’s disease (HD)	*S. cerevisiae*, *S. pombe*, *P. pastoris*, *K. lactis*	PolyQ-expanded huntingtin, protein aggregation, mitochondrial dysfunction, oxidative stress, protein quality control	Modeling polyQ aggregation; modifier screens; analysis of mitochondrial dysfunction and energy metabolism; identification of pathways involved in protein quality control	Robust aggregation models; scalable genetic and chemical screens	Lack of neuronal architecture and behavioral phenotypes	[83,84,85,86,87,88,89,90]

Abbreviations: AD—Alzheimer’s disease; PD—Parkinson’s disease; HD—Huntington’s disease; Aβ—amyloid-beta; APP—amyloid precursor protein; polyQ—polyglutamine; α-syn—alpha-synuclein.

**Table 3:** Comparative mitochondrial genetics toolkit across yeasts: transformation capability, HR readouts, and disease-oriented applications [117,118,119,120,121,122].

Species	Direct mt Transformation	HR Efficiency	Key HR Measurement	Disease Focus
*S. cerevisiae*	Yes (biolistic)	High	ARG8^m assay, cytoduction	mtDNA mutations
*S. pombe*	No	Moderate	Population sequencing (PHI)	Heteroplasmy
*Y. lipolytica*	No	Low-mod	Petite reversion	OXPHOS/lipids

Abbreviations: mtDNA—mitochondrial DNA; HR—homologous recombination; ARG8^m—mitochondrial arginine biosynthesis reporter; PHI—pairwise homoplasy index; OXPHOS—oxidative phosphorylation.

**Table 4:** Summary of mitochondrial dysfunctions, *Y. lipolytica* phenotypes, and human clinical correlates.

Mitochondrial Dysfunction	*Y. lipolytica* Phenotype	Human Clinical Correlates (Mitochondrial Disease)	References
Energy-metabolism impairment (low ATP)	No growth on non-fermentable carbon sources (glycerol/ethanol); reduced oxygen consumption/ATP synthesis	Leigh syndrome; mitochondrial myopathies (exercise intolerance, muscle weakness)	[146,152,153]
Oxidative stress (excess ROS)	Hypersensitivity to H_2_O_2_; increased ROS; mitochondrial fragmentation; reduced viability	Redox-imbalance-linked neurodegeneration (encephalopathies, neuronal damage)	[149,154]
Defective assembly/stability of respiratory complexes (I, IV)	Improper assembly and reduced complex activity (BN-PAGE/in-gel assays); heightened inhibitor sensitivity	Encephalopathies due to NUBPL/Ind1 (complex I) and COX deficiencies (complex IV)	[147]

Abbreviations: ATP—adenosine triphosphate; ROS—reactive oxygen species; BN-PAGE—blue native polyacrylamide gel electrophoresis; COX—cytochrome c oxidase; NUBPL/Ind1—complex I assembly factor; H_2_O_2_—hydrogen peroxide.

**Table 5:** Comparative evaluation of yeast models for MD research.

Organism	Strengths	Weaknesses	Applications/Diseases Studied	References
*Y. lipolytica*	It possesses complex I, making it ideal for investigating defects in this complex, and serves as a model for diseases such as Leigh syndrome. It is amenable to genetic modification and is an obligate aerobe.	Limitations include fewer genetic tools than in *S. cerevisiae* and its being a less widely used model.	Typical applications encompass complex I-related diseases, studies of respiratory-complex assembly, and pharmacological testing, including drug repurposing.	[125,146,147]
*S. cerevisiae*	The best-studied eukaryote, with an extensive repertoire of genetic tools; it serves as a principal model for mitochondrial DNA mutations and for investigating oxidative stress.	It lacks complex I and, being partially fermentative, is less dependent on respiration.	Research on mtDNA replication and segregation, and on reactive oxygen species (ROS) and energy homeostasis.	[145,148,149,150,151]
*S. pombe*	It is more closely related to human cells in terms of the cell cycle, possesses complex I, and is well-suited for studies of mitochondrial dynamics.	It is less widely used than *S. cerevisiae*, has a more limited repertoire of mutants, and its genetic toolkit is less developed.	Research focuses on mitochondrial dynamics (fusion, fission, inheritance), organelle stability, and mechanistic models of organelle division.	[141,151]

Abbreviations: MDs—mitochondrial diseases; mtDNA—mitochondrial DNA; ROS—reactive oxygen species.

**Table 6:** Comparative genetic tractability of yeast models used in cancer research.

Yeast Species	Genetic Tools/Approaches Described	Biological Processes Studied	Applications in Cancer Research	References
*S. cerevisiae*	Deletion collections; haploinsufficiency and homozygous profiling; synthetic lethality screens; genetic interaction mapping, CRISPR-based perturbations	Chromosome instability, DNA damage response, homologous recombination, checkpoint control, telomere maintenance	Identification of cancer-related genes; discovery of synthetic lethal interactions; functional validation of oncogenes and tumor suppressors	[20,156,157,158,159,163,164]
*S. pombe*	Targeted gene disruption; functional genetic assays	Cell-cycle regulation, checkpoint signaling, DNA repair	Functional characterization of conserved cancer-related pathways	[22,163,164,165]

Abbreviations: CRISPR—clustered regularly interspaced short palindromic repeats; DNA—deoxyribonucleic acid.

## Data Availability

No new data were created or analyzed in this study. Data sharing is not applicable to this article.

## Abbreviations

The following abbreviations are used in this manuscript:

AD	Alzheimer’s disease
ADo	Activation domain
Aβ	Amyloid-beta peptides
alg3	Asparagine-linked glycosylation protein 3 (Alg3 mannosyltransferase)
ALT	Alternative lengthening of telomeres
AMPK/Snf1	AMP-activated protein kinase/sucrose non-fermenting 1
AOX	Alternative oxidase
APH1	Anterior pharynx-defective 1 (γ-secretase subunit)
Arg	Arginine
Asa1	Asa1, subunit of the ASTRA/TTT complexes
ATM	ataxia-telangiectasia mutated
ATP	Adenosine triphosphate
BLM	Bloom syndrome protein
BN-PAGE	Blue native polyacrylamide gel electrophoresis
BRCA1	Breast cancer type 1 susceptibility protein
BRCA2	Breast cancer type 2 susceptibility protein
Cdk5	Cyclin-dependent kinase 5
CDKs	Cyclin-dependent kinases
CHO	Chinese hamster ovary
CIN	Chromosome instability
COX	Cytochrome c oxidase
CRISPR/Cas9	Clustered regularly interspaced short palindromic repeats/CRISPR-associated protein 9
DBD	DNA-binding domain
DNA	Deoxyribonucleic acid
DSBs	DNA double-strand breaks
ER	Endoplasmic reticulum
FMN	Flavin mononucleotide
FTDP-17	Frontotemporal dementia with parkinsonism linked to chromosome 17
GATOR2	GAP activity toward Rags complex 2
GFP-Aβ42	Green fluorescent protein–amyloid-beta 42 fusion
GPCRs	G protein-coupled receptors
GSK-3β	Glycogen synthase kinase 3 beta
HD	Huntington’s disease
HER2	Human epidermal growth factor receptor 2
HIF-1	Hypoxia-inducible factor 1
HIP	Drug-induced haploinsufficiency profiling
HOP	Homozygous profiling
HR	Homologous recombination
HTP	High-throughput morphological profiling
HTS	High-throughput screening
HTT	Huntingtin gene
IBD	inflammatory bowel disease
LBs	Lewy bodies
MAPT	Microtubule-associated protein tau gene
MDa	Megadalton
MDR	Multidrug resistance
MDs	Mitochondrial diseases
Mds1p	Yeast kinase Mds1 (GSK-3β ortholog involved in tau phosphorylation)
MOA	Mechanisms of action
Mkc7p	Yeast yapsin-like protease Mkc7
MSP	Multi-copy suppression profiling
mtDNA	Mitochondrial DNA
mTOR	mammalian target of rapamycin
NADH	Reduced nicotinamide adenine dinucleotide
och1	Outer chain protein 1 (α-1,6-mannosyltransferase Och1)
OXPHOS	Mitochondrial oxidative phosphorylation
PARK9	Parkinson’s disease 9 gene (ATP13A2)
PARP	Poly(ADP-ribose) polymerase
PD	Parkinson’s disease
PDR	Pleiotropic drug resistance
Pdr1p	Pleiotropic drug resistance transcription factor Pdr1
Pdr3p	Pleiotropic drug resistance transcription factor Pdr3
Pdr5p	Pleiotropic drug resistance ABC transporter Pdr5
PEN2	Presenilin enhancer 2 (PSENEN)
Pho85p	Cyclin-dependent protein kinase Pho85
polyQ	Polyglutamine
PP1	Protein phosphatase 1
PPIs	Protein–protein interactions
Rad51	Recombinase Rad51
Rad52	DNA repair protein Rad52
Rad55	Recombination mediator protein Rad55
RAS gene	Rat sarcoma oncogene family gene
rY2H	Reverse yeast two-hybrid
RNA	Ribonucleic acid
ROS	Reactive oxygen species
SARS-CoV-2	severe acute respiratory syndrome coronavirus 2
SCFA	Short-chain fatty acid
SGA	Synthetic genetic array
SGD	Saccharomyces Genome Database
SEACAT	Seh1-associated complex activating subcomplex
SL	Synthetic lethality
Snq2p	Multidrug efflux pump Snq2
Sup35	Translation termination factor eRF3 (Sup35)
TAA	Tumor-associated antigen
Tel1p	Tel1 protein (ATM homolog)
TOR	Target of rapamycin
tRNA	Transfer RNA
Tti1	Tel2-interacting protein 1
TTT	Tel2–Tti1–Tti2 complex
VLPs	Virus-like particles
Yap3p	Yeast aspartyl protease 3
YKO	Yeast knockout
YSD	Yeast surface display
Y3H	Yeast three-hybrid
Y2H	Yeast two-hybrid
α-syn	Alpha-synuclein
